# Prolonged Response to Osimertinib in EGFR-Mutated Metastatic Urothelial Carcinoma, a Case Report

**DOI:** 10.3390/curroncol31070298

**Published:** 2024-07-14

**Authors:** Sareen T. Ali, David J. VanderWeele

**Affiliations:** 1 Department of Medicine, Hematology/Oncology Division, Feinberg School of Medicine, Chicago, IL 60611, USA; 2 Robert H. Lurie Comprehensive Cancer Center, Northwestern University, Chicago, IL 60611, USA

**Keywords:** urothelial cancer, bladder cancer, EGFR, TKI, osimertinib, case report

## Abstract

A 48-year-old woman without obvious environmental risk factors was diagnosed with metastatic urothelial carcinoma harboring a mutation in *EGFR* typical of driver mutations for non-small cell lung cancer. Within a year, her cancer progressed on four standard therapies for urothelial cancer, including cancer in lungs, liver, bone, and brain. As fifth-line therapy, she received osimertinib, leading to a complete response in the brain and improvement elsewhere, and the cancer remained controlled for six months. Targeted therapy for rare driver mutations can be effective in urothelial cancer and should be considered prior to exhausting standard therapies.

## 1. Introduction

Metastatic urothelial cancer (mUC) is a lethal disease with limited treatment options and generally poor outcomes. For decades, platinum-based therapy was the mainstay of treatment for mUC, and it remains a treatment option today. From 2017 through 2022, there were several new approvals, but life expectancy remained 1–2 years. These approvals included multiple approvals for immunotherapy and the antibody–drug conjugates enfortumab vedotin (EV) and Sacituzumab govetecan (SG). Erdafitinib was also approved for patients with qualifying FGFR gene alterations, typically in *FGFR3*. For patients without *FGFR3* alterations, a standard treatment sequence was a platinum agent plus gemcitabine in the first-line setting, which was followed by maintenance avelumab if there was a response or by second-line therapy with pembrolizumab if there was no response. EV and SG were typically given in the third and fourth-line settings. The recent approval of EV plus pembrolizumab in the first-line setting has disrupted this sequence with no clear consensus on subsequent lines of therapy or sequence of therapy. Erdafitinib remains the only biomarker-selected therapy with an mUC-specific approval.

The epidermal growth factor receptor (EGFR) family regulates signal transduction pathways involved in cell proliferation. Mutations in EGFR have been associated with multiple cancer histologies and are an important target of anti-cancer therapies, most commonly in lung adenocarcinoma. Alterations in EGFR family members have also been described in urothelial carcinoma, but there are limited data on the use of EGFR-targeted therapies in this patient population. In this case report, we describe a woman with urothelial carcinoma harboring an EGFR mutation, metastatic to the lung, liver, bone, lymph nodes and brain. The cancer demonstrated a prolonged response to osimertinib as fifth-line therapy.

## 2. Case Report

The patient is a woman without a smoking history who presented at age 48 with increasing urinary frequency, urgency, nocturia, and pelvic pressure. Urine cytology revealed high-grade urothelial carcinoma, and cystoscopy showed multiple bladder masses. Pathology from the transurethral resection of bladder tumor (TURBT) confirmed high-grade papillary urothelial carcinoma with muscularis propria and vascular invasion. Staging scans revealed nodal, osseous, and pulmonary metastatic disease, stage IVB (pT2 cN2 cM1b).

The patient received first-line standard of care gemcitabine 1000 mg/m^2^ D1, 8 + cisplatin 70 mg/m^2^ D1 q21 days and underwent palliative radiation for osseous metastases. Restaging imaging after two cycles of therapy showed a progression of metastatic disease with multiple new osseous lesions and new nodal disease.

For second-line therapy, the patient participated in a clinical trial and received atezolizumab 1200 mg q21 days plus weekly recombinant human CYT107 (IL-7). Initial staging scans showed a minimal progression of osseous and nodal metastases. The patient continued in the trial through three cycles of therapy after which restaging scans revealed multifocal disease progression with new osseous metastases in the spine, progressing pulmonary metastases, new metastatic lymphadenopathy, and new hepatic metastases. She also experienced clinical progression and had a prolonged hospitalization for pain control attributed to osseous metastases.

After her pain was adequately controlled, the patient received standard EV 1.25 mg/kg D1, 8, 15 q28 days as third-line therapy. Restaging scans after two cycles of therapy demonstrated an overall response with resolution of hepatic metastases, decreased tumor burden in lungs and lymph nodes, and stable osseous metastases, and she also experienced improvement in pain. However, after two additional cycles of therapy, restaging scans demonstrated a progression of disease with increased size and number of osseous, pulmonary, and hepatic metastases. The patient then received sacituzumab govitecan (SG) 10 mg/kg D1, 8 q21 days as fourth-line therapy. After four cycles, she was found to have progression in pulmonary nodules and a new small parietal lobe metastasis measuring. SG was discontinued.

**Figure 1:**
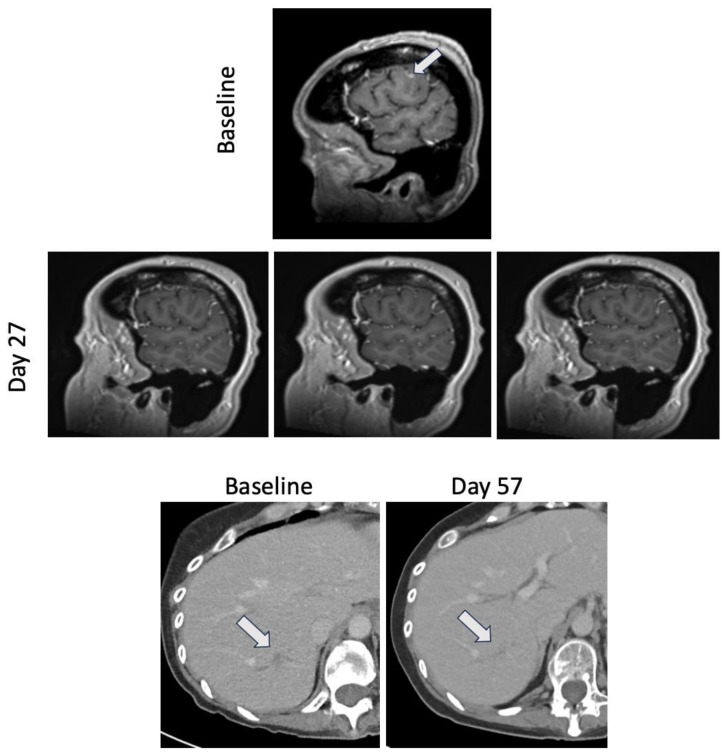
Response to osimertinib. Magnetic resonance images showing brain metastasis at baseline prior to starting osimertinib and at day 27 of daily osimertinib therapy (**top** panel). Computed tomography images showing liver metastasis at baseline prior to starting osimertinib and at day 57 of daily osimertinib therapy (**bottom** panel).

Molecular testing using cancer gene panel sequencing had been performed on plasma (a 105 gene panel) and the diagnostic biopsy tissue (a 648 gene panel), both of which revealed a gain-of-function *EGFR* exon 19 indel (L747_E749del). No other targetable alterations were identified. Frameshift or missense mutations were also identified in *ARID1A*, *CDKN1A*, *ELF3*, *RHOA*, and *SPOP*. Staining of her original TURBT showed HER2 2+ staining. Given only modest HER2 expression, targeting EGFR was prioritized over targeting HER2. Coverage for off-label therapy was denied by her insurance, but osimertinib was provided by the manufacturer. After stopping SG, the patient switched to osimertinib 80 mg daily to target the EGFR alteration. Repeat MRI brain imaging four weeks after initiating osimertinib showed resolution of the parietal lobe metastasis (Figure 1). Disease evaluation at two months showed improvement in hepatic, pulmonary and nodal metastatic disease with stable osseous metastasis (Figure 1). Repeat disease evaluation at four months showed continued stable disease. However, six months after starting osimertinib, she had disease progression with new osseous metastatic lesions, new hepatic metastasis, progressing nodal metastasis, new malignant effusion, and new parietal lobe metastasis.

With progression of disease, the patient switched from osimertinib to pembrolizumab while appealing for insurance coverage for off-label therapy targeting HER2. However, the patient died from progressive mUC within 2 months of progression on osimertinib. 

## 3. Discussion

Urothelial carcinoma (UC) is characterized by high rates of invasion, early metastasis, and a high mortality rate. It is the eighth leading cause of cancer death in the United States with 16,710 deaths expected in 2023 [1,2]. Muscle-invasive bladder cancer (MIBC) comprises two thirds of invasive UC and has high morbidity and mortality. Currently, standard treatment for UC is neoadjuvant chemotherapy followed by cystectomy with options for adjuvant systemic immunotherapy or chemotherapy [2]. Unfortunately, many patients relapse, and half of the patients treated with definitive therapy die within five years [3]. Immune checkpoint inhibitors have emerged as an additional treatment option in advanced UC with two new chemoimmunotherapy regimens (EV + pembrolizumab and gemcitabine + cisplatin + nivolumab) showing improvement in overall survival over standard systemic therapy in the first-line metastatic setting, yet median overall survival for metastatic disease continues to be less than 3 years [4,5]. Thus, there is a great need for new targeted therapies for UC, especially treatments tailored to specific tumor genomics.

**Figure 2:**
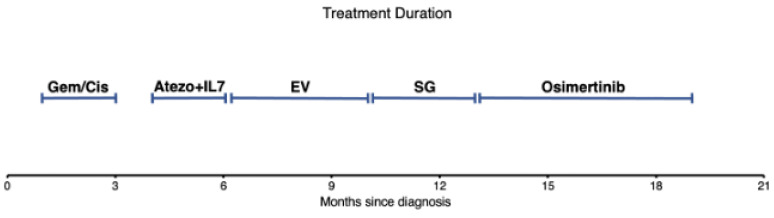
Length of treatment duration for each systemic therapy.

We describe here a patient with metastatic urothelial carcinoma with EGFR exon 19 indel which progressed rapidly on standard therapies for urothelial carcinoma but demonstrated prolonged response to osimertinib, which is a third-generation tyrosine kinase inhibitor targeting EGFR. She received four lines of therapy prior to initiating osimertinib with a median duration of 73 days of treatment prior to progression for standard therapy vs. 176 days for osimertinib (Figure 2). The cancer responded to osimertinib with a resolution of multiple small lesions, including a brain metastasis. The cancer did not progress until six months after starting therapy, which was a longer duration than on any of the four prior regimens. To our knowledge, this is the first report of osimertinib for UC, and the patient received significant benefit from it. Thus, this case reflects the importance of molecular testing in providing treatment options in UC and the utility of targeting rare driver mutations that predict sensitivity to targeted therapy in other cancer histologies. It supports the case that therapy targeting likely driver alterations should be covered by insurance and other payers and should be incorporated into earlier lines of therapy.

The patient received two cycles of gemcitabine + cisplatin, three cycles of immunotherapy, four cycles of enfortumab vedotin, and four cycles of sacituzumab govitecan for a median treatment duration of 73 days prior to progression. She had disease control for 176 days on osimertinib.

### 3.1. Targets of UC Therapies

Targeted therapies have become increasingly important in UC/MIBC. For example, about 20% of UC harbor alterations in fibroblast growth factor receptor 3 (FGFR3), which has resulted in targeted FGFR-inhibitor therapies like erdafitinib receiving accelerated approval from the Food and Drug Administration (FDA) based on objective response rates ~40% in early trials [6,7]. Another potential treatment target is the ErbB family: a class of tyrosine kinases consisting of EGFR (ErbB1), HER2 (ErbB2), HER3 (ErbB3), and HER4 (ErbB4). The molecular characterization of UC as part of The Cancer Genome Atlas (TCGA) and other studies has revealed a high incidence of ErbB family mutations and an over-expression in UC with alterations occurring in up to 20–30% of UC [8].

### 3.2. EGFR

EGFR overexpression in UC is correlated with high invasion, recurrence, and tumor grade [9]. EGFR is highly expressed in up to 72.2% of UC, and EGFR amplification, missense and in-frame mutations occur in approximately 5% of cases; most of these are gene amplification [10,11,12,13,14]. High rates of overexpression and alteration mark EGFR as a possible treatment target in UC.

EGFR-targeting tyrosine kinase inhibitors (TKIs) have been commonly used to target and treat EGFR-positive non-small cell lung carcinoma (NSCLC) with differential sensitivity of TKIs to different EGFR mutations [9]. However, clinical data examining TKI treatment in UC or MIBC is limited and mixed. A first-generation TKI, erlotinib, has shown promise as neoadjuvant therapy in MIBC, but its effects were limited, with only 25% of patients achieving pT0 on surgical pathology. Specific EGFR mutations in patients were unknown [15]. A second-generation TKI, afatinib, is approved for metastatic NSCLC with specific EGFR exon 19 deletion. One study found that patients with EGFR-positive UC did not benefit from afatinib, but the patient population did not have EGFR exon 19/21 mutations for which afatinib is approved [16]. The third-generation TKI osimertinib has been shown to inhibit urothelial carcinoma cell proliferation and colony formation in preclinical studies, but it has not been studied clinically [17]. Our case supports the use of osimertinib in UC with EGFR exon 19 alteration as our patient derived more durable benefit from osimertinib than from each of four prior lines of therapy. Although currently molecular analysis is indicated for evaluation for FGFR mutations, rare incidental findings that predict response to available therapies should also be incorporated into management plans. 

### 3.3. HER2

Alterations in HER2, another member of the ErbB family, are another area of interest for emerging targeted therapy, especially antibody–drug conjugate (ADC) therapies in UC. While HER2 alterations are most commonly found in breast cancer, among solid tumors, UC has the third highest rate of HER2 overexpression. Anti-HER2 therapies such as trastuzumab and TKIs (apatinib, lapatinib) have shown mixed results [18,19]. 

The first developed anti-HER2 ADC was trastuzumab emtansine (TDM-1), which showed promising preclinical data in vitro and in vivo, though clinical results for solid tumors other than breast cancer have been more disappointing [20]. Trastuzumab duocarmazine is another ADC which has shown partial response specifically in cases of heavily pretreated metastatic HER2 + UC [21]. Multiple novel anti-HER2 agents are being developed, including disitamab vedotin, which has shown a promising response rate in advanced or metastatic HER2 + UC [22], and ongoing trials are evaluating trastuzumab deruxtecan for an array of solid tumors that express HER2 (NCT04482309). While routine molecular testing for HER2 expression in UC is not a part of current practice, studies with anti-HER2 therapy suggest that there may be benefit to targeting HER2, and thus molecular testing may prove useful for patients with advanced UC when determining options for therapy.

## 4. Conclusions

Our case demonstrates the utility in routine molecular testing in UC and targeting rare driver genomic alterations that may be more common in other types of cancer (EGFR in NSCLC). Our patient’s UC with EGFR exon 19 alteration responded well to osimertinib as a fifth-line therapy with the resolution of some metastasis and prolonged response relative to prior therapies. Unfortunately, with little data on each rare mutation, it is difficult to obtain insurance approval and coverage for such therapies. It is unknown if the patient would have had a more durable response if osimertinib was used as an earlier line of therapy. Patients with similar genetic alterations may also benefit from osimertinib, and hopefully case series and case reports such as this one can add to the rationale of using off-target therapy for rare driver alterations.

Targeted therapies have shown significant potential in the treatment of advanced UC, but more targets and treatment combinations are needed. In the case of NSCLC, numerous different targeted therapies exist and can be tailored to identified driver alterations discovered in molecular testing. To reach this degree of treatment specialization for patients with UC, routine molecular testing will be a key tool to identify treatment targets, develop new therapies, and improve the use of current therapies, resulting in better individualized treatment plans and outcomes for our patients.

## Data Availability

De-identified data is available upon request from the Corresponding Author.
